# Rapid assessment of clinical severity for salmonellosis cases via protein family domain analysis and machine learning

**DOI:** 10.20935/acadmolbiogen7776

**Published:** 2025-06-30

**Authors:** Aakash Bhattacharyya, Sudip Panday, David Ussery

**Affiliations:** 1 Little Rock Central High School, Little Rock, AR 72202, USA.; 2 Department of Biomedical Informatics, University of Arkansas for Medical Sciences, 4301 W. Markham St., Slot 782, Little Rock, AR 72205, USA.

**Keywords:** Salmonella, machine learning, bacterial genomics, Pfam domains, Domains of unknown function (DUFs), severity prediction

## Abstract

*Salmonella* is a common pathogen, infecting more than a million people yearly. Rapid assessment of clinical case severity is essential for improving patient outcomes and optimizing healthcare resources. Advancements in genome sequencing technologies have enabled the analysis of bacterial genomes from many clinical cases, opening up new opportunities for precise and timely diagnosis. This study proposes a genome-based framework for identifying critical *Salmonella* cases before the onset of critical symptoms and facilitating early medical intervention. By leveraging protein family (Pfam) domains as the representation for genomic data, the complex genetic profiles of *Salmonella* cases are simplified into interpretable features. The severity levels of cases were investigated through rigorous data analysis, resulting in a set of 70 Pfam domains that could be potentially used as biomarkers. Machine Learning was employed to assess the predictive power of the curated Pfam biomarkers, achieving high accuracy (~93%) in sorting cases into critical, moderate, and mild categories. The results demonstrate the efficacy of the proposed approach. This framework highlights the potential of using bacterial genomic data in clinical decision-making, opening the window for timely personalized interventions for *Salmonella* infection management.

## 1. Introduction

### 1.1. Purpose

This study aims to discover bacterial genomic-based biomarkers indicating the clinical severity of *Salmonella,* which medical doctors can use to provide personalized early intervention.

### 1.2. Literature review

Foodborne illnesses significantly burden society, with *Salmonella* being one of the top four leading causes. Every year, almost 1 in 10 people worldwide fall ill with a foodborne disease, and 33 million years of healthy life are lost [1]. *Salmonella* is categorized as a diarrheal disease, which can be life-threatening, with nearly half a million children dying from it annually, and the annual number of infections is much larger [1]. Most cases of Salmonellosis (clinical manifestations of *Salmonella*) are mild and require little to no treatment, but sometimes, it can be severe and life-threatening [1]. Salmonellosis has the potential to cause various life-threatening symptoms, such as typhoid, sepsis, meningitis, and more. These complications often arise when the bacteria penetrate beyond the intestines, a progression that is heavily influenced by the factors encoded in the bacterium’s genome [2]. Salmonellosis begins with generic initial symptoms, typically diarrhea, which develops 1–3 days after exposure [3].

The subsequent clinical severity of the disease (which develops after 4–7 days) depends primarily on toxin and virulent factors present in the bacteria’s genome and host factors [3]. When severe, early treatment can be the difference between life and death. “Prompt medical treatment can be lifesaving because of the high mortality and morbidity associated with [*Salmonella*]” [4]. The unpredictability of *Salmonella* cases makes determining the best treatment difficult for doctors. The economic impact of *Salmonella* infections is just as devastating, with the U.S. alone estimating around $3.7 billion in loss of productivity annually [5]. The higher the severity of the case, the higher the financial burden due to various factors such as surprise of onset. This burden is also growing because of the prevalence of antibiotic resistant strains of *Salmonella*, which complicates treatment and can extend hospital stays and recovery times [2]. Recently, extensively drug-resistant (XDR) strains of *Salmonella* Typhi have been reported to be resistant to first-line and second-line antibiotics [6]. The inability to assess the severity of cases until the onset of critical symptoms delays treatment for life-threatening cases of *Salmonella*. The more quickly doctors can identify life-threatening cases, the faster they can provide the necessary line of treatment, saving lives, reducing complications, and improving healthcare productivity by reducing financial burdens. For example, the case–fatality ratio of typhoid fever, a dangerous complication caused by *Salmonella*, can be as high as 30%. However, early intervention can reduce the case–fatality ratio to lower than 1% [7]. Determining a method for quickly assessing the clinical severity of *Salmonella* cases and specifically identifying life-threatening cases is of utmost importance for advancing the treatment of *Salmonella*.

### 1.3. Proposed implementation framework

**Figure 1 •:**
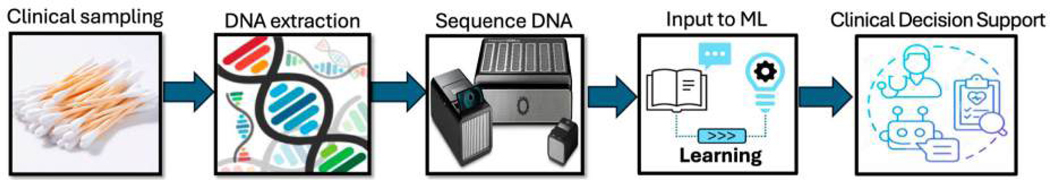
A potential implementation framework for severity prediction of Salmonella infections, based on genome sequences.

Because *Salmonella* cases tend to start with the same generic symptoms, critical cases are identified after the onset of critical symptoms, resulting in unnecessary hospital stays, complications, or death. While there have been attempts by previous researchers to potentially identify information about *Salmonella* before the onset of critical symptoms through culturing, serotyping, and more [5], these efforts are time-consuming (taking days) and not fast enough to be applicable to cases quickly for early treatment. A more recently established way to identify life-threatening cases of *Salmonella* in time to avoid the critical symptoms is through sequencing with third-generation technology, such as Oxford Nanopore flow cells, which can give sequence reads from *Salmonella* genomic DNA within 30 min. With technological advancements in genome sequencing and the development of next-generation genome sequencing tools, obtaining a whole-genome sequence (WGS) is now a timely and affordable option, and it is possible to sequence organisms with large, multi-billion bp (Gbp) genomes, such as humans, within a day [8]. Oxford Nanopore sequencing technologies have revolutionized microbial genomics [9]. Whole-genome sequencing for *Salmonella* genomes (~5 Mbp) from clinical isolates can be obtained within 30 min, at a cost of below $20 per sample [10], using purified chromosomal DNA from a monoculture [11]. Further, metagenomics sequencing of fecal material and clinical isolates can be carried out within a few hours, without the need for a purified culture [12], as shown in Figure 1.

The proposed framework is based on the bacterial genome sequence. Doctors could leverage the knowledge of biomarkers by obtaining *Salmonella* genome sequences from clinical cases. The machine learning model or SSP (Severe *Salmonella* Predictor) would be able to rapidly assess the biomarkers present and provide a severity prediction, allowing doctors to provide essential early intervention if required.

## 2. Methods and materials

### 2.1. Data acquisition

*Salmonella* genomes with clinical case metadata were obtained from the PATRIC Database [13] on 2024 Aug 11. At the time of obtaining the data, only approximately 40,000 genomes were available. However, not all the genomes were usable for the research. Criteria for narrowing down the genomes into ‘eligible genomes’ were set. The first criterion was that the genome must belong to the species of *Salmonella*. The following criterion was that the host of the clinical case had to be human, and the genome sequence had to be readily available. The PATRIC database marks each genome as either ‘Good quality’ or ‘Poor quality.’ The next criteria set required the genomes to be designated as ‘Good quality.’ Lastly, as clinical cases of *Salmonella*, the age, gender, and case outcome of the host had to be listed and interpretable. For interpretability, if the age of the host had no unit, it was eliminated due to it being unclear. Ultimately, only 500 ‘eligible genomes’ were left after all the criteria were set.

### 2.2. Data stratification

As per the criteria set in the previous step, each eligible genome listed a host gender, host age, and host case outcome; the cases were categorized and then stratified based on these values. The severity category requirements were divided into three categories: critical (in need of emergency medical attention); moderate (infected and sick but not an emergency); and mild (no treatment required). For categorizing clinical outcomes into severity categories, critical cases were identified as cases requiring emergency attention: bacteremia, sepsis, meningitis, and typhoid. Moderate cases were identified as any cases where treatment would be required, likely in the form of fluids but not emergency attention: urinary tract infection, dysentery, intestinal complications, gastroenteritis, respiratory infection, and abscess. Lastly, mild cases were identified as any case where the patient needed rest and likely did not require the assistance of a doctor: asymptomatic, mild diarrhea. After each case was categorized based on a severity level (critical, moderate, or mild), the age of the host was categorized into Groups 1–6: Group 1 ranged from 0 to 2 years, Group 2 ranged from 3 to10 years, Group 3 ranged from 11 to 24 years, Group 4 ranged from 25 to 40 years, Group 5 ranged from 41 to 65 years, and Group 6 encompassed 66 years and above. Lastly, the host’s gender was categorized as male or female.

### 2.3. Data selection

The next step was stratifying the data based on the previously set categories. Specific categories of data, such as mild cases for patients aged 10–24, had a proportionally higher number of cases. However, the research sought an equal representation of all the data combinations. So, the cases were stratified based on severity categories, age groups, and gender, covering all combinations, and cases were randomly selected from each, ensuring representation from all combinations. Due to time and resource constraints, only 99 isolates were randomly selected. The genomes from these 99 isolates resulted in more than 1.5 million Pfam domain records. This is because each genome contains more than four thousand proteins, and each protein could contain several Pfam domains; in rough numbers, 100 genomes × 4000 proteins/genome x 4 domains per protein equals about 1.6 million. The complete set genomes of 500 available isolates would have resulted in almost 8 million Pfam (protein family) domain records, although the number of unique Pfam domains is much smaller (there are only about 24,000 unique Pfam domains in the current version). Although there are more than 2500 different ‘types’ (Serovars) of *Salmonella*, only a few are human-specific, and these are divided into categories: typhoidal (causing typhoid fever) and non-typhoidal [14]. The typhoidal type of *Salmonella* are mainly characterized with the Typhi and Paratyphi strains. The Enteritidis and Typhimurium serotypes are the most common pathovars [15]. However, Enteritidis is less pathogenic because fewer cases turn severe [15]. This increases the value of finding *Salmonella*-specific biomarkers that can potentially differentiate between the serovars. In terms of severity level, the overall distribution of the selected cases was as follows: approximately 31% were mild, 31% were moderate, and 38% were critical. The age group breakdown of the selected cases is as follows: 16% for Groups 1, 3, and 5; 19% for Groups 2 and 6; and 13% for Group 4. The gender split for the selected cases was 52% male and 48% female.

### 2.4. Obtaining the genomic information

The 99 selected genomes were downloaded from GenBank on 2024 Aug 11, and the protein sequences were extracted from the genome assemblies.

#### 2.4.1. Protein family (Pfam) domains

**Figure 2 •:**
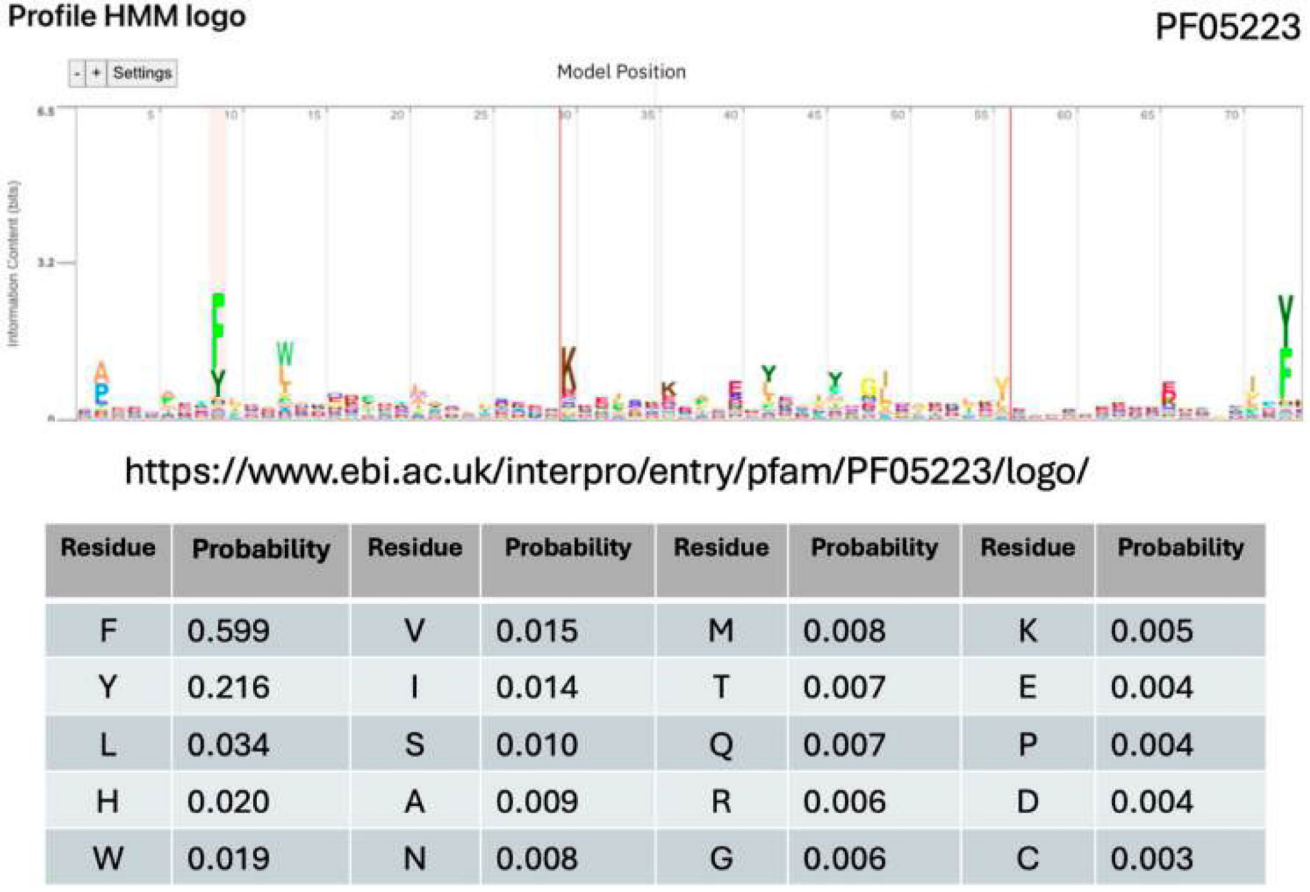
Example protein sequence logo of Pfam domain PF05223 from InterPro: probability per placement for a Pfam is calculated depending on sequence alignment comparison to the input sequence.

*Salmonella* holds a considerable amount of genetic information in its DNA. Each *Salmonella* genome codes for over 4000 proteins [16], and each protein can be made from as little as ten amino acids to thousands of amino acids. Comparing whole protein sequences can be time-consuming and complicated. The main reason for this is that proteins can have the same function (for example, the same protein domains) but different amino acid sequences [17]. As more genomes have been sequenced, protein diversity has exploded, and the conservation of full-length amino acid sequences of the ‘same’ proteins can be quite low, as shown in the sequence logo in Figure 2. This figure represents the alignment of a structural domain [PF05223] from a large set of proteins that provide resistance to the antibiotic methicillin. Outbreaks of methicillin-resistant *S. aureus* (MRSA) strains commonly occur, and the mecA gene is responsible for encoding a protein, penicillin-binding protein 2a (PBP2a), which provides resistance to methicillin. The important point in Figure 2 is that very few of the amino acids in this protein are strongly conserved, making it difficult to compare the raw protein sequences accurately. There are only a few key amino acids necessary for function, and the rest can vary. Therefore, a way to efficiently and accurately compare protein sequences from acquired whole genome sequences must be found.

One approach provides a potential solution: protein family (Pfam) domains. The Pfam database, first introduced in 1997 [18], is a growing resource for inferring the functionality of protein domains, and in 2022, it was merged with InterPro [19]. Pfam does not categorize proteins directly based on the primary full-length protein sequences but by their conserved protein functional domains. Protein domains are defined as distinct functional units in a genome [20]. Aside from making a comparison across proteins much more feasible, using domains significantly simplifies the data, making it easier to interpret. Pfams are a much more robust way of interpreting genomic information [21]. Proteins can be converted into their respective protein family domains (Pfams) through a program called HMMER. HMMER works on a Hidden Markov Model and uses sequence alignments of domains as inputs. There are over 20,000 Pfams as of HMMER version 3.3.2, and a profile of each domain has been created [19]. When entered, a protein is compared to the various profile HMMs of Pfams and given a value (E-value) reflecting the probability of a match. If the probability hits a critical value, it is considered a match, and the protein is identified as part of that Pfam [22]. Many proteins have multiple domains.

To reflect how well a protein’s amino acid sequence matches with the profile HMM of PF05223, an E-value is given. If the E-value reaches a significant value, suggesting a hit, that protein will be categorized with the PF05223 functionality, implying the protein has a role in Beta-Lactam antibiotic resistance through the gene mecA, for example. Looking at Figure 2, only a handful of amino acids are conserved across the more than eight thousand proteins used to build this model; most of the amino acids can vary without strongly affecting the function of the domain. For example, position 9 is highlighted, which is usually phenylalanine (F) or Tyrosine (Y); this would be considered an essential position of proteins with this function. As indicated by the chart, 60% of more than 8000 proteins used to build PF05223 have the amino acid phenylalanine in the highlighted position, whereas the position before (position 8) on the Pfam model has no strongly conserved amino acids in the proteins with the functionality of PF05223. This indicates that it is not an essential position for determining PF05223 functionality, demonstrating the robustness of Pfams to mutations in areas not important for function.

#### 2.4.2. Conversion to Pfams

HMMER was used to convert protein sequences to their respective Pfam ‘hits.’ HMMER works on a profile Hidden Markov Model (HMM); in other words, it identifies query sequences that match one of the many profiles in its database. This can be used for Pfam identification. The file of all the profile HMMs of Pfams was set as a database in HMMER, and it was able to convert the whole genome into Pfam ‘hits.’ All randomly selected genomes were converted to Pfams.

After obtaining the Pfam files from the genomes, they were transformed, and all the Pfam files were loaded into a SQL server (1.5+ Million Records).

## 3. Biomarker verification through machine learning

To evaluate the efficacy of the biomarkers, a machine learning model (named the SSP—*Salmonella* Severity Predictor) was developed using the Python programming language and the XGBOOST-supervised machine learning algorithm. XGBoost (Extreme Gradient Boosting) is a powerful, open-source machine learning algorithm that implements gradient boosting, a method used for building predictive models by combining the predictions of multiple weak learners, often decision trees, in an iterative manner. It is known for its speed, efficiency, and ability to scale well with large datasets, making it popular for both regression and classification tasks [23].

In this research, the following XGBoost parameters were used: params = {“max_depth”: 6, “objective”: “multi:softmax”, ”num_class”: 3, “tree_method”: “hist”, “eta”: 0.3, ‘seed’: 0, ‘min_child_weight’: 1}. Along with the above, a boosting cycle of 1000 and 20 early stopping rounds were used. Once the genomes were run through HMMER, a dataset with the isolates and their associated Pfams was run against the curated Pfam biomarkers list to create a new dataset. The Isolate and Pfam biomarker combinations were used to create a dataset which was eliminated for duplicate combinations. Then, this dataset was split randomly to generate training and testing datasets.

**Figure 3 •:**
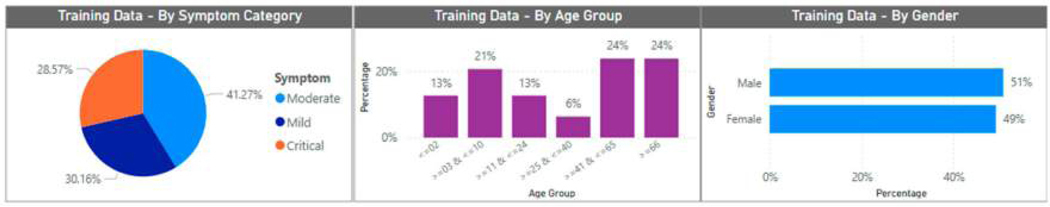
Training set distribution between case categories.

**Figure 4 •:**
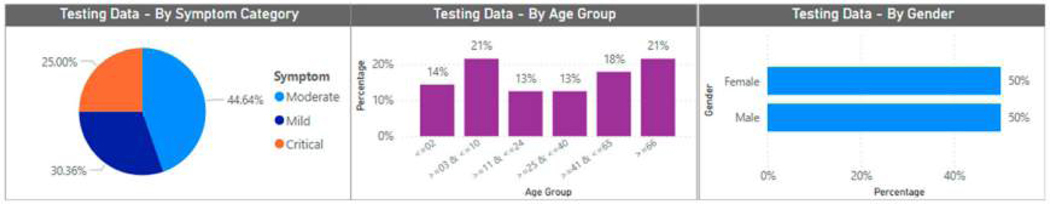
Testing set distribution between case categories.

If the SSP accurately predicted the clinical severity of *Salmonella* cases solely based on the biomarkers curated, this would validate the findings. The distribution of symptom categories, age groups of hosts, and gender of hosts for the training set data are shown in Figure 3. A nearly even distribution is demonstrated. The distribution of symptom categories, age groups of hosts, and gender of hosts for the testing set data are shown in Figure 4. Again, a nearly even distribution is demonstrated.

Both the training set and testing sets have nearly perfect splits between the symptom categories, age groups of the hosts, and gender of the hosts. The training set and testing sets also have nearly identical distributions.

## 4. Results

### 4.1. Serovar distribution

**Figure 5 •:**
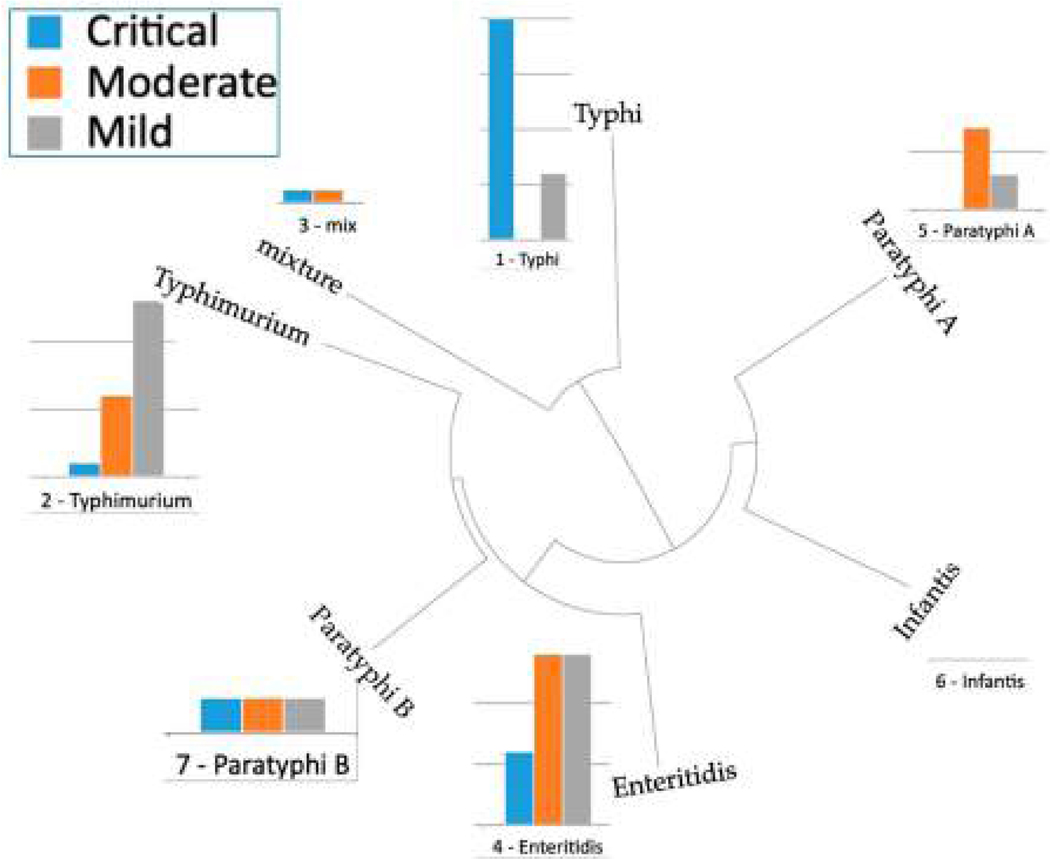
The serotype distribution of the selected cases and the associated severity levels.

Figure 5 shows the distribution of *Salmonella* infection levels (critical, moderate, mild) amongst the 99 randomly selected genomes, distributed across a set of seven different *Salmonella enterica* subspecies enterica phylogroups. Shown in the figure is the medioid genome for each phylogroup, constructed from a set of 4000 complete *Salmonella* genomes, using the Mash program, as described previously [24]. The distribution of severity cases is not random, with the ‘critical’ severity associated genomes clustering more closely with genomes of the *Salmonella* Typhi serotype, whilst genomes for the ‘moderate’- and ‘mild’-severity genomes clustered with the *Salmonella* Paratyphi and *Salmonella* Enteriditis serotypes, and the ‘mild’ cases clustered with S. Typhimurium genomes, as shown in Figure 5. Note that none of the 99 genomes clustered with the Infantis phylogroup.

### 4.2. Biomarker discovery

After loading the proteins from 99 randomly selected genomes into a SQL server, Pfam presences were compared between critical, moderate, and mild cases to determine causation and curate a list of biomarkers for severity assessment of *Salmonella.* The two criteria used to curate the biomarkers were exclusivity and high correlation. Exclusivity was characterized as Pfams exclusive to multiple critical isolates, multiple moderate isolates, or multiple mild isolates. High correlation was generally characterized as Pfams that tended to be highly prevalent in critical cases and have only a few appearances in mild or moderate cases, Pfams that tended to be highly prevalent in moderate cases and have only a few appearances in critical or mild cases, or Pfams that tended to be highly prevalent in mild cases and have only a few appearances in critical or moderate cases. After obtaining this list of preliminary biomarkers for severity assessment, any Pfams clearly unrelated to pathogenicity based on their Pfam entry were eliminated. Domains of unknown function (DUFs) were kept. In the end, 70 Pfam biomarkers were left. Based on their Pfam entry, the functionality of each Pfam biomarker as broad categories, such as bacterial motility or antibiotic resistance, were noted. We found 15 Pfam biomarkers that were enriched in the genomes associated with critical infections, as shown in Table 1. These include two antibiotic resistance genes (“CAT” and “TetR” domains, encoding resistance to chloroamphenical and tetracycline, respectively), and a set of domains involved in the Type 6 secretion complex (“T6SSS”). Further, there is a domain associated with flagella activation (FapA, a putative DNA-binding domain as part of a gene of unknown function). We also found 30 Pfam domains enriched in moderate cases (Table 2), and a different set of 25 domains enriched in the mild cases (Table 3).

### 4.3. DUF (Domains of Unknown Function) prediction

Of the 70 biomarkers identified, 21 were classified as ‘Domains of Unknown Function’ (DUFs). This is 30% of the identified biomarkers. To help predict their function, the *Salmonella* proteins with the DUF domains were examined in the String database [25, 26], where factors such as protein–protein interactions, gene occurrence, gene neighborhood, gene fusion, and more were considered to predict the potential function of proteins having domains of unknown function. The predicted functionalities of the DUFs are listed in Table 4. A substantial portion (6 of the 21 DUFs) of the DUFs still had unidentifiable functionalities. However, it should be noted that two of the DUFs found in the ‘critical’ case genomes are part of the Type 6 secretion system, which can inject toxins into the host cell [27]. Further research is needed to investigate these domains’ roles in *Salmonella* severity.

### 4.4. Pfam biomarker results

**Figure 6 •:**
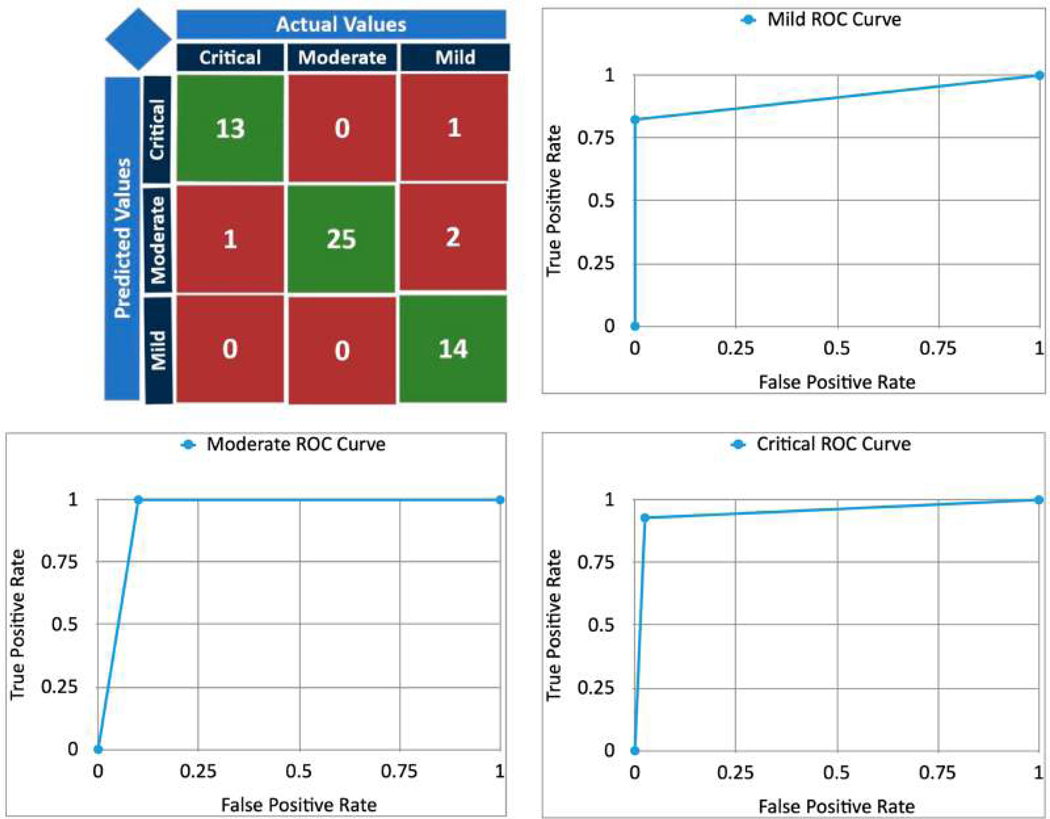
Confusion matrices for critical case, moderate case, and mild case predictions, along with their respective ROC curves.

**Figure 7 •:**
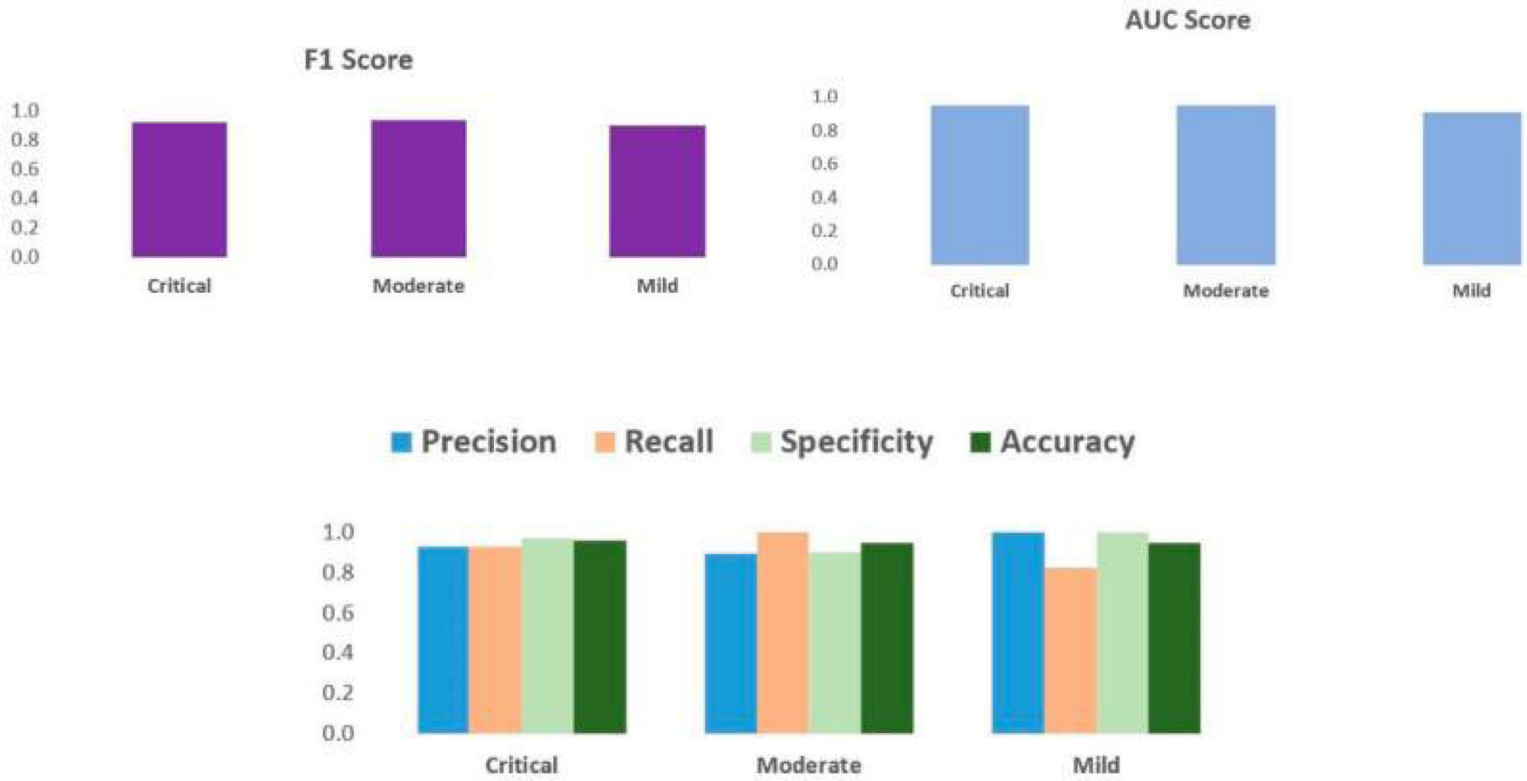
Graphs for F1 scores; AUC scores; and precision, accuracy, recall, and specificity scores for critical, moderate, and mild case predictions.

**Figure 8 •:**
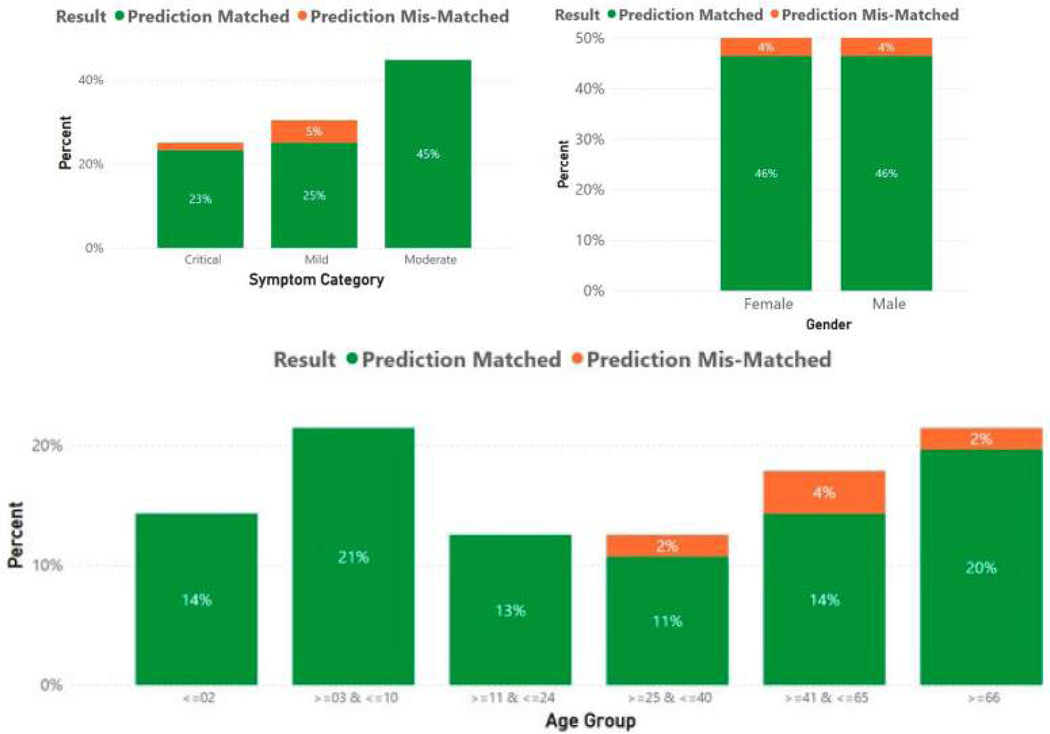
Overall accuracy of the SSP based on biomarkers across the different age groups, symptom categories, and genders.

The machine learning model based on the biomarkers correctly predicted 52/56 [93%] of the cases correctly. The Receiver Operating Characteristic (ROC) curve compared the True Position Rate and False Positive Rate and was graphed for every level of severity, as shown in Figure 6. All the ROC curves demonstrate the high efficacy of the model [28]. The AUC (Area Under the Curve) was calculated for the ROC curves graphed in Figure 6. The statistics of precision, recall, and specificity were calculated for the predictions for every severity level, and the F1 score was calculated for each of the severity levels, with precision and recall scores having equal weightage, as shown in Figure 7. The accuracies of the model across each of the severity levels, age groups, and genders were checked, and the results are shown in Figure 8 and Table 5.

## 5. Discussion

With the advent of third-generation sequencing technology, such as Oxford Nanopore flow cells, it is now possible to do metagenomics with very short sequencing times. For example, 10 min can identify viruses, [29] and pathogens from blood samples [30]. With a few hours’ sequencing time, at enough depth, nearly complete bacterial genomes can be obtained. The computational analysis described in this paper starts with a *Salmonella* genome sequence, which can be quickly assembled from long reads, with strain-level resolution in less than 20 min [31]. The SSP [Salmonella Severity Predictor] demonstrated a high accuracy (~93%) of predicting the clinical severity of *Salmonella* cases based solely on the presence of the 70 biomarkers found. In other words, clinical decision-making would be significantly improved if the proposed framework was applied in real clinical settings based on these 70 biomarkers. Figure 6 illustrates the confusion matrices for predicting critical, moderate, and mild cases individually. The SSP predicted critical, moderate, and mild cases with high accuracies based on the biomarkers found. Also in Figure 6, the ROC curves for each symptom category are near-perfect, demonstrating promising results. Figure 7 demonstrates the F1 scores; AUC (Area Under the Curve) for the ROC curves; and precision, recall, and specificity scores calculated for the prediction of each symptom category. The F1 scores and AUC value were near-perfect for the SSP predictions of critical, moderate, and mild cases. F1 scores and AUC scores are metrics of the model’s overall performance, in addition to the overall accuracy calculated in Figure 6. Precision, recall, and specificity were calculated as well. Precision is defined as the proportion of correct positive predictions. The SSP had precision scores of 1, 0.89, and 0.93 for mild, moderate, and critical cases, respectively. Recall score is defined as the proportion of positive instances that were identified correctly. The SSP had recall scores of 0.82, 1, and 0.93 for mild, moderate, and critical cases, respectively. Specificity is defined as the proportion of negative instances that are calculated correctly. The SSP had specificity scores of 1, 0.9, and 0.98 for mild, moderate, and critical cases, respectively.

**Figure 9 •:**
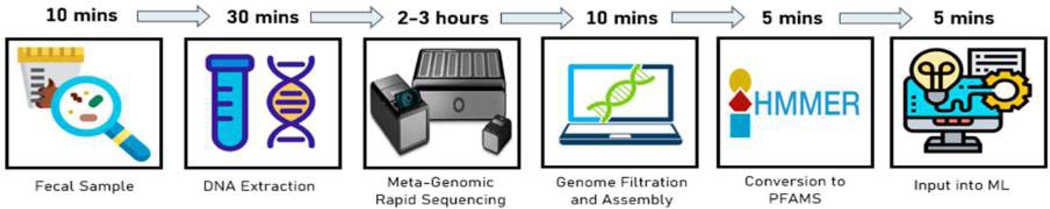
Potential step-by-step process for hospital implementation.

All performance metrics portrayed promising results on the SSP, and the biomarkers’ predictive capabilities and are reiterated in Figure 9. In Figure 8, the accuracy of the SSP based on the biomarkers was monitored across the host’s age groups, symptom categories, and genders. There were no gender imbalances in predictive capabilities. The SSP, however, demonstrated higher predictive capabilities for younger hosts. The SSP also demonstrated perfect accuracy in predicting the severity of moderate cases. Given the high accuracy of the model, in principle, the model could be implemented in hospital settings much faster than current methods in a streamlined process of genome acquisition through meta-genomic sequencing, followed by conversion to Pfams, and ultimately input into the model, as outlined in Figure 9. After the expense of the fixed cost of setting up, the per-patient expense would be approximately a few hundred dollars for the whole sequencing process.

## 6. Conclusions

In principle, the SSP can predict critical cases accurately and quickly, before onset of critical symptoms, based on the genomic information of the *Salmonella* case. Early prediction of critical cases leads to earlier intervention and treatment, which could save thousands of lives. Therefore, the SSP would help to save lives, reduce complications and hospital stay, and cut down the massive financial burden on patients and the healthcare system.

## 7. Future Outlook

In the future, the SSP will be expanded to include a larger volume of genomes and a vast amount of more complicated factors, such as age-progression-based Pfam factors and Pfam count factors. The model will also be assessed on related diarrheal diseases besides *Salmonella*, such as *E. coli* or *Campylobacter*. Additionally, the DUFs identified as clinical biomarkers for severity in *Salmonella* should be further studied to understand its role.

## Figures and Tables

**Table 1 •:** The 15 Pfam biomarkers associated with critical cases of *Salmonella* infection.

PFAM biomarkers by name	PFAM accession	Rationale
CAT	PF00302	Critical
DHC_N2	PF08393	Critical
DUF2254	PF10011	Critical
DUF2345	PF10106	Critical
DUF4019	PF13211	Critical
DUF4123	PF13503	Critical
DUF724	PF05266	Critical
Lambda_Kil	PF06301	Critical
Put_DNA-bind_N	PF06971	Critical
T6SS_FHA_C	PF20232	Critical
T6SS_VasJ	PF16989	Critical
T6SS_Vgr	PF13296	Critical
TetR_C_4	PF08359	Critical
VasI	PF11319	Critical
FapA	PF20250	Critical

**Table 2 •:** The 30 Pfam biomarkers associated with moderate cases of *Salmonella* infection.

PFAM biomarkers	PFAM accession	Rationale
Ac45-VOA1_TM	PF20520	Moderate
DDE_Tnp_IS66_C	PF13817	Moderate
DndE	PF08870	Moderate
DSPc	PF00016	Moderate
DUF1524	PF07510	Moderate
DUF2523	PF10734	Moderate
DUF2577	PF10844	Moderate
DUF3302	PF11742	Moderate
DUF4424	PF14415	Moderate
DUF5677	PF18928	Moderate
EcoRII-C	PF09019	Moderate
DUF5680	PF18931	Moderate
DUF772	PF05598	Moderate
EcoRII-N	PF09217	Moderate
Fic_N	PF13784	Moderate
FlxA	PF14282	Moderate
FRG	PF08867	Moderate
GIIM	PF08388	Moderate
HEPN_RES_NTD1	PF18870	Moderate
HGSNAT_cat	PF07786	Moderate
IrmA	PF18673	Moderate
DUF1380	PF07128	Moderate
DUF3482	PF11981	Moderate
DUF932	PF06067	Moderate
PemK_toxin	PF02452	Moderate
Plasmid_stab_B	PF10784	Moderate
PsiA	PF06952	Moderate
PsiB	PF06290	Moderate
S_4TM	PF18159	Moderate
TraH	PF22351	Moderate

**Table 3 •:** The 25 Pfam biomarkers associated with mild cases of *Salmonella* infection.

PFAM biomarkers	PFAM accession	Rationale
7tm_7	PF08395	Mild
Abhydrolase_4	PF08386	Mild
Apolipoprotein	PF01442	Mild
DarT	PF14487	Mild
DUF1194	PF06707	Mild
DUF1494	PF07379	Mild
DUF2220	PF09983	Mild
DUF2787	PF10980	Mild
DUF5937	PF19361	Mild
Glyco_hydro_32C	PF08244	Mild
Glyco_hydro_32N	PF00251	Mild
Glyco_hydro_36C	PF16874	Mild
Glyco_hydro_36N	PF16875	Mild
GP17	PF17420	Mild
Melibiase	PF02065	Mild
Ndr	PF03096	Mild
Ntox34	PF15606	Mild
OMP_b-brl_2	PF13568	Mild
Phage_P22_NinX	PF10765	Mild
Phenol_Hydrox	PF02332	Mild
SIR2_2	PF04574	Mild
Xre_MbcA_ParS_C	PF09722	Mild
ToxN_toxin	PF13958	Mild
RepC	PF06504	Mild
YqeC	PF19842	Mild

**Table 4 •:** DUFs (sorted numerically) with predicted functionality from STRING database.

DUF724	Still unknown	Critical
DUF772	Enzyme Function	Moderate
DUF932	Predicted Lipoprotein	Moderate
DUF1194	ABC transporter	Mild
DUF1380	Still Unknown	Moderate
DUF1494	Cleavage protein	Mild
DUF1524	Predicted Ligand Binding Protein	Moderate
DUF2220	Still Unknown	Mild
DUF2254	Predicted Membrane Protein (Mechanochannel)	Critical
DUF2345	Predicted Type 6 Secretion System (Vgr)	Critical
DUF2523	Still Unknown	Moderate
DUF2577	Phage Protein	Moderate
DUF2787	Superfamily transporter	Mild
DUF3302	Predicted Inner membrane protein	Moderate
DUF3482	Ligand	Moderate
DUF4019	Still Unknown	Critical
DUF4123	Predicted Type 6 Secretion System (Vgr)	Critical
DUF4424	Effux Pump	Moderate
DUF5677	Enzyme Function	Moderate
DUF5680	Still Unknown	Moderate
DUF5937	Still Unknown	Mild

**Table 5 •:** Statistical results for the performance of the *Salmonella* Severity Predictor (SSP).

Symptom category	True positive	True positive	False positive	False negative	Acccuracy	Precision	Recall	Specificity	F1	AUC
Critical	13	39	1	1	96%	0.93	0.93	0.98	0.93	0.95
Moderate	25	27	3	0	95%	0.89	1.00	0.90	0.94	0.95
Mild	14	38	0	3	95%	1.00	0.82	1.00	0.90	0.91

## Data Availability

Data supporting these findings are available within the article, at https://doi.org/10.20935/AcadMolBioGen7776, or upon request.
